# Circular RNA circRNA_0067934 promotes glioma development by modulating the microRNA miR-7/ Wnt/β-catenin axis

**DOI:** 10.1080/21655979.2022.2033382

**Published:** 2022-02-25

**Authors:** Yunlong Pei, Hongying Zhang, Kongye Lu, Xiaojia Tang, Jialing Li, Enpeng Zhang, Jun Zhang, Yujia Huang, Zhijie Yang, Zhenggang Lu, Yuping Li, Hengzhu Zhang, Lun Dong

**Affiliations:** a Department of Clinical Medicine, Dalian Medical University, Dalian, Liaoning, China; b Department of Neurosurgery, Clinical Medical College of Yangzhou University, Yangzhou, Jiangsu, China; c Department of Image, Clinical Medical College of Yangzhou University, Yangzhou, Jiangsu, China; d Department of Rehabilitation, Clinical Medical College of Yangzhou University, Yangzhou, Jiangsu, China; e Department of clinical science, Ross University School of Medicine (RUSM), Bridgetown, Barbados; f Department of Neurosurgery, Fudan University, Yangzhou, Jiangsu, China

**Keywords:** circRNA_0067934, circRNAs, miR-7, glioma

## Abstract

Glioma, one of the most prevalent malignant tumors, is well-known for its poor prognosis and low survival rate among patients. As a type of non-coding RNA, circular RNAs (circRNAs) play a significant role in tumor progression. However, the function and role of circRNAs in glioma development remain unclarified. In our experiments, the relative expression level of circRNA_0067934 and miR-7 in glioma tissue was detected by qRT-PCR, and specific gene knockdown was mediated by siRNA and miRNA-inhibitor. Dual-luciferase reporter assay was carried out to determine whether miR-7 successfully targeted circRNA_0067934. Also, CCK-8 and Transwell were performed to evaluate the malignant behaviors of glioma tissues. Western blotting and immunofluorescence were used to evaluate relative protein expression levels. The results of qRT-PCR indicated that circRNA_0067934 was over-expressed in glioma tissues, and down regulation of circRNA_0067934 reduced the tumor progression by inhibiting cell proliferation, invasion, and migration. The relative expression level of miR-7 was significantly reduced in glioma tissues, which showed a negative association with the expression of circRNA_0067934. CircRNA_0067934 could tagete the miR-7 to regulate progression of glioma cell. In addition, the Wnt/β-catenin signaling pathway might involve in down stream regulation of circRNA_0067934 and miR-7. In conclusion, our results revealed that circRNA_0067934 regulates glioma cells progression by targeting miR-7/ Wnt/β-catenin axis.

## Graphical Abstract

**Figure:**
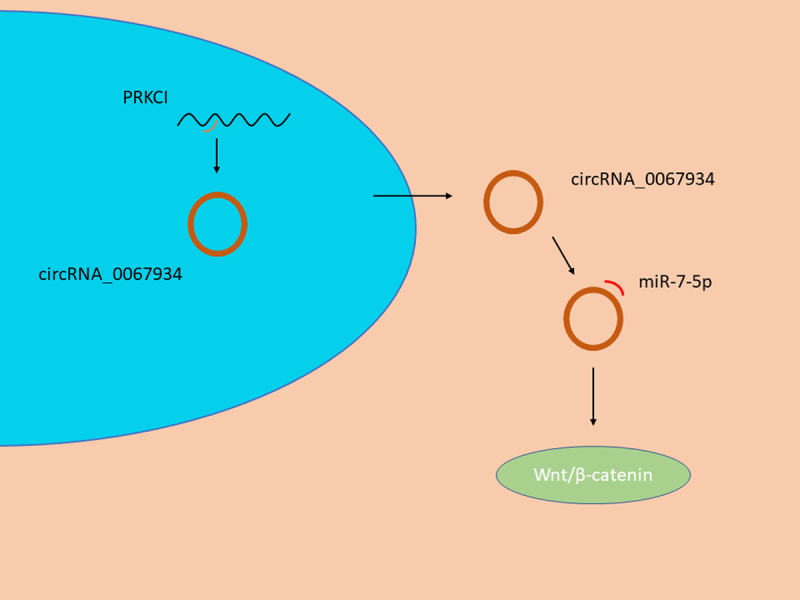

## Introduction

Glioblastoma multiforme (GBM) is one of the most aggressive and common adult cancers in the central nerves system. Its prognosis is usually poor and patients suffer from GBM often have low survival rate [1]. Despite improvements in standard treatments and clinical guidance, including maximal safe surgical resection, radiation therapy, and drug treatment by temozolomide, the median survival rate of GBM patients remains poor [2–4]. Thus, new therapeutic strategies should be explored, with a goal to improve the prognostic outcome of GBM patients. The molecular mechanism of glioma, including significant gene and proteins, has been extensively studied, leading to many newly evolved treatment strategies.

As a type of non-coding RNAs, circRNAs provide a crucial role in gene regulation. As production of back-splicing of exons or lariat introns, circRNAs usually present covalent bonds of the 3’ and 5’ ends, and are abundant, evolutionally conserved in kind of cells [5,6]. The molecular structure of circRNAs is a continuous loop and thus circRNAs are not easily degraded, ensuring its stability when compared to that of linear RNAs [7,8]. Circular RNAs are further categorized into three types: exon circRNAs, circular intron RNAs, and exon-intron circRNAs [9–11]. Studies in recent years shown that Circular RNA performs its regulatory function in a variety of ways, including modulating the transcription process by influencing the linear splicing compete of pre-mRNA to regulate related genes expression [12], miRNA sponging [7,8,13], generating functional proteins [13], and circRNA–protein interactions [14,15]. Recent studies shown that circRNAs, as oncogenes, are significantly associated with cancer development and biological function. For example, circRNA_0000392 can promote colorectal cancer cells’ proliferation and invasion by targeting the miR-193a-5p/PIK3R3/AKT axis [16]. Also, down-regulated expression of circDCUN1D4 can promote metastasis and regulate glycolysis of lung adenocarcinoma through binding the ternary complex of circDCUN1D4/human antigen R/thioredoxin-interacting protein RNA-protein [17]. For glioma, previous studies have increasingly identified evidence that circRNAs promote proliferation and predict overall survival as a novel biomarker [18,19]. As for glioma, previous studies provided evidence that circRNAs promote glioma cells proliferation and thus can be used to monitor overall prognosis as a novel biomarker [18,19]. However, there is still scant evidence showing correlation between the specific circRNA, circRNA_0067934, and glioma [20].

As another type of non-coding RNA, microRNAs are significantly associated with cell function in various types of cells. Previous studies suggested that aberrant expression of miRNAs up- or down- regulated the development and progression of cancers [21,22]. miRNAs can target other molecules, including long non-coding RNA, circRNA, and protein, to regulate apoptosis, invasion, proliferation, and metastasis of cells [7,8,13,16,17,20,22]. As for glioma, multiple evidence indicated that miRNAs play a critical role in tumor development [21,22].

As a tumor suppressor, miR-7 involves in glioma development and progression. Evidence shown that down-regulation of miR-7 can accelerate the process of epithelial–mesenchymal transition, therapeutic resistance, and invasion behavior [23]. Though increasing evidence have shown the function of miR-7, the underlying mechanism of miR-7 and its effect on glioma remain unclear.

In the present study, we aimed to identify a potential molecular mechanism between circRNA_0067934 and miR-7, and the subsequent effects on glioma cells. We proposed that circRNA_0067934 could sponge miR-7 to regulate biological function via wnt/β-catenin axis in glioma cells. This study enriches our understanding of circRNAs on glioma progression and provides a clinical reference for prognosis monitoring in patients with glioma. This study enriches our understanding of circRNAs on glioma progression and provides a clinical reference for prognosis monitoring in patients with glioma.

## Materials and methods

### Glioma tissues collection

In this study, the experimental protocols were approved by the Medical Ethics Committee of the Yangzhou School of Clinical Medicine of Dalian Medical University (2021ky138-1). A total of 16 clinical tissue samples were collected from Northern Jiangsu People’s Hospital of Jiangsu Province from January 2020 to July 2021, including 8 glioma tissue samples and 8 brain tissues samples with non-glioma diseases. The diagnosis of glioma was confirmed by pathology. All the patients signed informed consent. After resection, all glioma tissues were snap-frozen in liquid nitrogen and stored at − 80 ℃ immediately.

### Cell culture and transfection

HEK 293 T cell lines, U87 glioma cell lines, and U251 glioma cell lines were generous gifts from Prof. Yufu Zhu (Xuzhou Medical University). In this study, high glucose DMEM (Hyclone, Solarbio, China) was used to culture cells, which contains 1% penicillin/streptomycin (Gibco, Carlsbad, CA, USA) and 10% fetal bovine serum (CLARK, CLARK Bioscience, USA). All cells were cultured at 37℃ in a humidified chamber with 5% CO2.

Several knockdown materials were purchased from Sangon Biotech (Shanghai, China). Including small interfering RNAs (si-hsa-circ-0067934), miR-7 inhibitors (anti-miR-7), and their negative controls. According to the manufacturer’s instructions, RNATransMate (Sangon Biotech, Shanghai, China) was applied to cell transfection.

### qRT-PCR assays

Total RNAs were extracted from tissue samples and cultured cells using Trizol reagent (Invitrogen, USA). The miRNA-cDNA Synthesis Kit (CW2141S, CWBIO, China) and circRNA fluorescence quantitative PCR Kit (GS0201-1, GENESEED, China) were applied to synthesize cDNA. Then qRT-PCR analyses were carried out to detect the relative expression level of miR-7 and circRNA_0067934, by using miRNA qPCR Assay Kit (CW2142S, CWBIO, China) and circRNA fluorescence quantitative PCR Kit following the manufacturer’s protocol. U6 and GAPDH were carried out as internal references for miRNAs and circRNAs, respectively. The relative expression level of circRNAs and miRNAs was calculated by the method of 2^–ΔΔCT^. The sequences of primers of circRNA, miRNA, GAPDH, and U6 were shown in supplement Table S1. Additionally, the reverse primer was provided by miRNA qPCR Assay Kit. The forward primer of U6 was purchased from (Sangon Biotech, Shanghai, China).

### CCK-8 assay

CCK-8 kit (Dojindo, Shanghai, China) was applied to evaluate the viability of glioma cells in different groups. Cells were plated in 96-well plates at a density of 1 × 10^3^ cells/well. After incubation for 24 hrs, CCK-8 solution (10 μl) was added into each well to the final working volume of 100 μl and incubated for 1.5 hrs. The absorbance at 450 nm wavelength was evaluated and recorded at different time points (12 hrs, 24 hrs, 48 hrs, and 72 hrs).

### Transwell assay

Cell migration assay was carried out by transwell chamber (Corning, New York, NY) by using a polyethylene terephthalate cell culture chamber with an 8 μm pore size. Specifically, in the upper chamber, a total of 5 × 10^4^ cells were plated and cultured with serum-free medium, of which the volume in the upper chamber was 300 μl. 600 μl of 10% FBS medium was added to the lower chamber. After incubating for 24 hrs at 37℃, cells were fixed with 4% paraformaldehyde for 15 mins, stained with Crystal Violet Staining Solution for 10 mins, and washed with phosphate buffer saline (PBS). The migrated cells were observed and photographed by an inverted microscope.

Cell invasion assays were performed by using penetrating 8-μm pore size polyethylene terephthalate membrane (Corning, New York, NY). Before the invasion assays started, Matrigel was pre-coated into the upper chamber and incubated. Glioma cells were added into the upper chamber and 600 μl culture medium with 10% FBS was added into the lower chamber. After incubation, the cells were fixed with 4% paraformaldehyde for 15 mins and stained with Crystal Violet Staining Solution for 10 mins. The invasion cells were photographed by an inverted microscope.

### Immunofluorescence (IF)

After transfection, glioma cells were washed with PBS and fixed with 4% paraformaldehyde for 15 mins. Then, 1% Triton X-100 (Beyotime, China) was used to permeabilize fixed cells for 10 min at room temperature. After that, fixed cells were blocked with 5% BSA for 10 mins at room temperature. The cells were incubated with rabbit polyclonal anti-beta catenin antibody (1:200, HuaBio, China) overnight at 4°C. After washing three times by PBS, cells were incubated with Donkey Anti-Rabbit IgG H&L preadsorbed (1:500, ab150064, Alexa Fluor 594, Abcam, UK) for 2 hrs at room temperature. Then DAPI (Beyotime, China) was used to stain the cell nuclei for 15 mins. Inverted fluorescence microscope (Zeiss, Germany) was used to observe and take photos.

### Western blotting

The different groups of glioma cells were lysed on ice by RIPA buffer (CW2333, CWBIO, China) adding protease inhibitors. Subsequently, protein concentration was identified by Micro BCA Protein Assay Kit (CW2011, CWBIO, China). Equivalent amounts of protein from different groups were resolved through 10% SDS-PAGE (Epizyme Biotech), transferred onto PVDF membranes (Millipore, Billerica, USA), and blocked with 5% skim milk. Primary antibodies were incubated overnight at 4°C, including rabbit polyclonal anti-beta catenin antibody (1:5000, HuaBio, China), rabbit polyclonal anti-WNT1 antibody (1:5000, HuaBio, China), and rabbit anti-Tubulin beta antibody (1:5000, affinity, China). Next, secondary antibodies (1:1000; Millipore, USA) were incubated for 1 hr.

### Dual luciferase reporter assay

The luciferase assay was performed by Promega Dual-Luciferase system. The plasmid psiCHECK2 was constructed to contain circ_0067934 (wild type, WT) or mutant sequences (MT). According to the grouping requirements, HEK293T cells were transfected with circ_0067934 (WT), circRNA_0067934 (MT), miR-7-mimic, or miR-7-NC. Promega Dual-Luciferase system was used to measure the activities of firefly luciferase and renilla luciferase.

### Statistical analysis

GraphPad Prism v8.01 (GraphPad, La Jolla, CA) was used to analysis all experimental data. and. Student’s t test or one-way ANOVA test was used to determine statistical significance. The data was expressed as mean ± SD. *P*< 0.05 was considered statistically significant.

## Results

The purpose of this study was to identify the function of circRNA_0067934 on glioma cells. We hypothesized that circRNA_0067934 promotes glioma development by modulating the miR-7/ Wnt/β-catenin axis. Several assays were carried out to find the regulation mechanism of circRNA_0067934 and miR-7, including qRT-PCR, CCK-8 assay, Transwell assay, dual-luciferase reporter assay, immunofluorescence assay, and Western blotting assay.

### CircRNA_0067934 was upregulated in glioma

**Figure 1.**
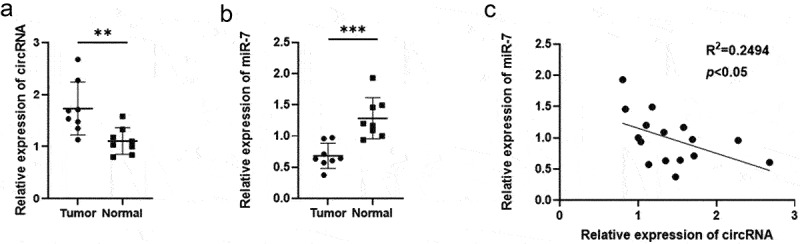
The expression level of circRNA_0067934 and miR-7in glioma tissues. (a) The relative expression of circRNA_0067934 was detected by qRT-PCR in glioma tissues and normal tissues. **p< 0.01 versus normal group. (b) The relative expression of miR-7 was detected by qRT-PCR in glioma tissues and normal tissues. ***p < 0.001 versus normal group. (c) The expression level of circRNA_0067934 was significantly negatively associated with the expression level of miR-7. R^2^ = 0.2494, *p*< 0.05.

To identify the potential role of circRNA_0067934, qRT-PCR was performed to detect the expression level of circRNA_0067934 in glioma tissue and normal tissue, and basic characteristics of glioma patients are shown in Table 1. The results indicated that circRNA_0067934 was significantly up-regulated in glioma tissues (Figure 1a, *p*= 0.007).

**Table 1.** Basic characteristics of glioma patients

Number	Gender	Age	Tumor size	WHO	Relative expression of circ_0067932	Relative expression of miR-7-5p
1	female	54	7*8*5	IV	2.277	0.959
2	male	51	5*5*5	IV	1.694	0.975
3	female	63	3.5*3.5*2.5	III	1.537	0.644
4	female	52	5*5*5	II	1.134	0.573
5	male	48	5*5*5	IV	2.680	0.607
6	female	44	6*5*5	IV	1.714	0.711
7	male	43	6*6*8	II	1.351	0.634
8	male	76	4*5*6	IV	1.478	0.375

### Effect of circRNA_0067934 on glioma cells’ viability, invasion, and migration by targeting wnt/β-catenin signaling pathway

**Figure 2.**
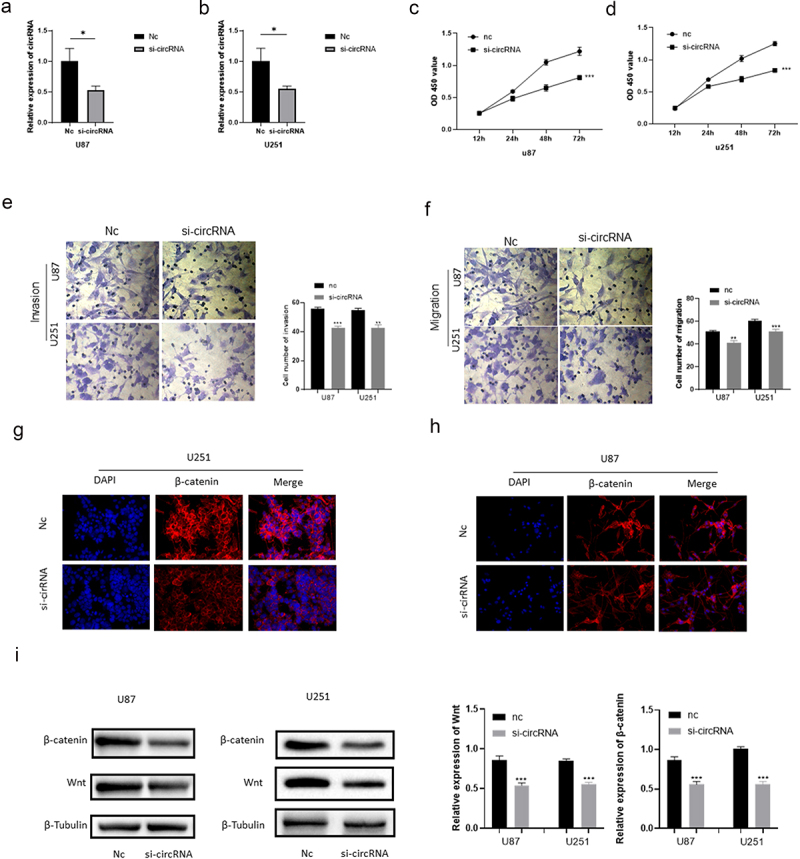
Effect of circRNA_0067934 on viability, invasion, and migration. (a) The expression level was down regulated in the siRNA group in U87 cells. *p*< 0.05 versus nc group. (b) The expression level was down regulated in the siRNA group in U251 cells. *p< 0.05 versus nc group. (c and d) The cell proliferation was determined by CCK-8 assay. In the siRNA group, the ability was down regulated. ***p < 0.001 versus nc group. (e and f) Transwell assay was performed to evaluate the invasion and migration. Scale bars = 20 μm. (g and h) The IF assay was used to detect the β-catenin expression. Scale bars = 20 μm. (i) The WB assay was used to determine wnt and β-catenin expression.

The siRNA of circRNA_0067934 was constructed and transfected into glioma cell lines U87 and U251 to evaluate the function of circRNA_0067934. The relative expression of circRNA_0067934 for siRNA group was significantly down-regulated in U87 or in U251 cells (Figure 2a,b). For cell viability, the results of CCK-8 assay suggested that cell viability was significantly down-regulated after transfecting the siRNA (Figure 2c,d). Transwell assay was performed to evaluate the ability of cells’ invasion and migration. As shown in Figure 2e,f, the amount of glioma cells was reduced in the siRNA group. Immunofluorescence assay suggested that β-catenin protein was down-regulated in the siRNA group in U87 and U251 cells (Figure 2g,h). Besides, the results of Western blotting indicated that wnt protein and β-catenin protein were down-regulated after down-regulating circRNA_0067934 (Figure 2i). In summary, these results demonstrated that down-regulation of circRNA_0067934 reduced the viability, invasion, and migration in glioma cells by modulating the wnt/β-catenin signaling pathway.

### MiR-7 was down-regulated in glioma tissues

QRT-PCR assay was used to detect the relative expression level of miR-7 in glioma tissue and in normal tissues. As shown in Figure 1b, miR-7 was significantly down-regulated in the glioma tissues (p = 0.0006). Besides, the expression level of miR-7 was negatively correlated to the expression level of circRNA_0067934 (R^2^ = 0.2494, *p*= 0.0489).

### CircRNA-0067934 sponges miR-7

**Figure 3.**
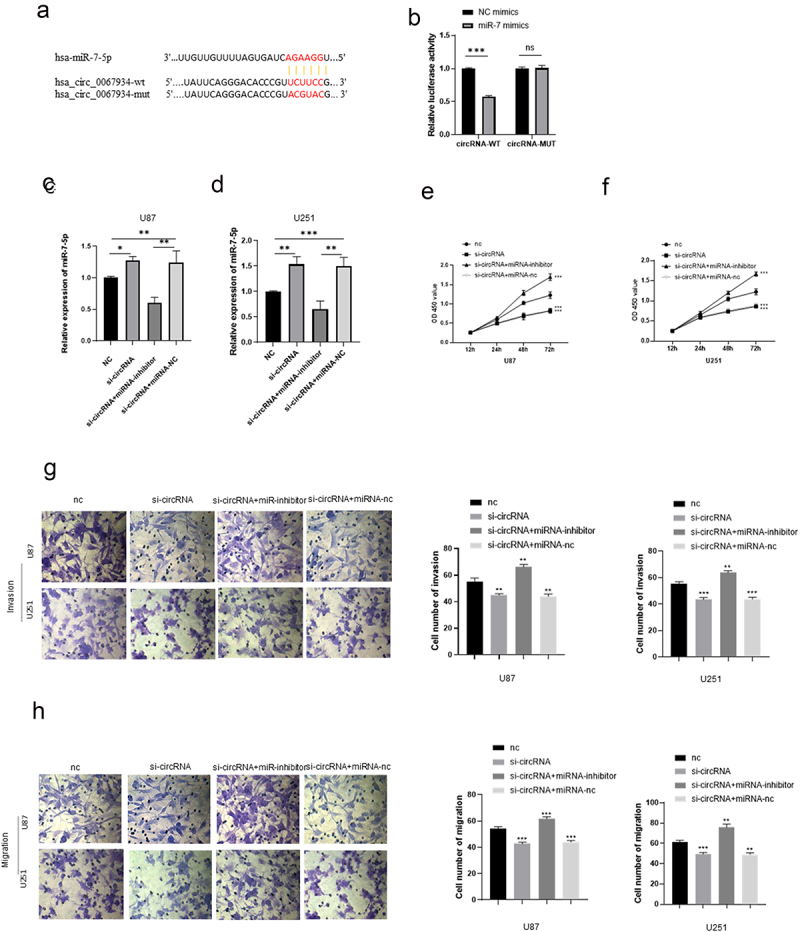
CircRNA_00679934 regulated glioma cells’ function by targeting miR-7 wnt/β-catenin axis. (a and b) The results of dual luciferase reporter assay demonstrated that circRNA_0067934 significantly targeted miR-7. ***p< 0.001. (c and d) The relative expression of miR-7 in different groups. * *p*< 0.05, **p< 0.01, ***p < 0.001. (e and f) The cell proliferation was determined by CCK-8 assay in U87 cells and U251 cells. ***p < 0.001. (g and h) Transwell assay was performed to evaluate the invasion and migration. Scale bars = 20 μm.

Dual-luciferase reporter assay was performed to evaluate the relationship between the miR-7and circRNA_0067934. The results showed that circRNA_0067934 significantly targeted miR-7 (Figure 3b, *p*< 0.05). After knockdown of circRNA_0067934, the relative expression level of miR-7 was significantly up-regulated in U87 and U251 cells. In addition, knockdown of miR-7 and circRNA_0067934 reduced the expression of miR-7, which partially reversed the effect of circRNA_0067934.

### circRNA _001783 targets miRNA-7 to regulate glioma progression by modulating wnt/β-catenin signaling pathway

**Figure 4.**
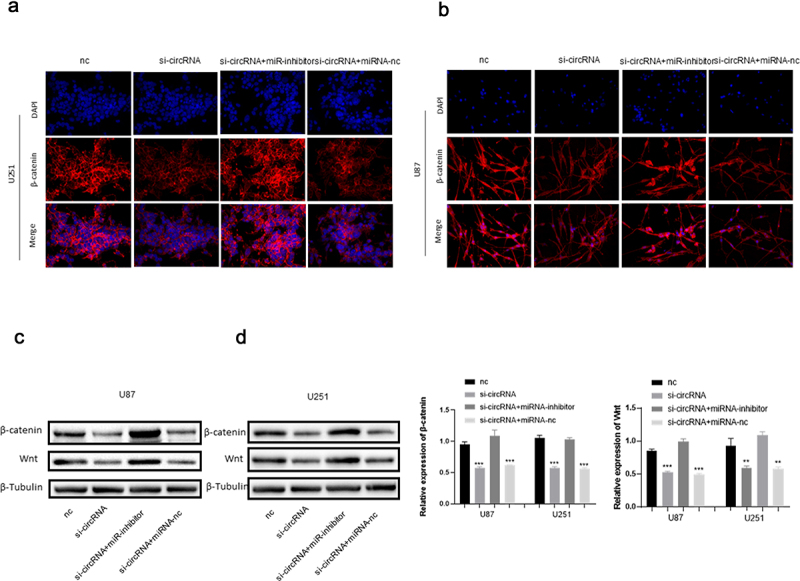
(a and b) The IF assay was used to detect the β-catenin expression. Scale bars = 20 μm.(c and d) The WB assay was used to determine wnt and β-catenin expression.

To further assess the function of circRNA_0067934 and miR-7, several other assays were performed, including CCK-8 assay, Transwell assay, Western blotting assay, and Immunofluorescence assay. The CCK-8 assay suggested that the cell viability was significantly reduced in the knockdown circRNA_0067934 group, and inhibition of miR-7 significantly reversed the effect (Figure 3e,f). As for cell invasion and migration, the results of Transwell assay indicated that down-regulation of miR-7 accelerated the invasion and migration behavior in U87 and U251 glioma cells (Figure 3g,h). Immunofluorescence and Western blotting assays suggested that wnt/β-catenin signaling pathway was regulated by circRNA_0067934 and miR-7. As shown in Figure 4, the protein expression level of wnt and β-catenin was down-regulated in the si-circRNA group and up-regulated in the si-circRNA and miR-7-inhibitor group. In summary, the above results suggested that circRNA_0067934 accelerated glioma progression by sponging miR-7 and modulating wnt/β-catenin signaling pathway.

## Discussion

In recent years, with the development of high-throughput sequencing technology, numerous circRNAs were discovered. CircRNA, as a novel biomarker, plays a significant role in tumor development and progression by regulating downstream factors [18–20]. A growing body of evidence suggests that circRNAs are involved in the malignant behaviors in glioma cells, promoting their proliferation, invasion, migration, apoptosis, and drug resistance [18,19,24]. However, the underlying mechanism remains not fully investigated. In this study, we identified that circRNA_0067934 was significantly up-regulated in the glioma tissues and it affects the malignant behaviors of glioma cells.

Functional analysis was performed to evaluate the effects of circRNA_0067934 on glioma cells. In this part, we reduced the expression level of circRNA_0067934 by using siRNA in order to evaluate the changes. The results indicated that the malignant behaviors of glioma cells were down-regulated in siRNA group, including cell proliferation, invasion, and migration. Therefore, circRNA_0067934 is significantly associated with development and progression of glioma cells.

Subsequently, the potential molecular mechanism of circRNA_0067934 on regulation of glioma cells was explored. Previous studies indicated that abnormal expression of miRNA was detected in various cancer cells and its abnormal expression regulated biological functions of those cells [21–23]. Previous studies also demonstrated that miR-7-5p is down-regulated in lung cancer cell lines and in NSCLC tissues, leading to suppression of cell proliferation, invasion, migration, and metastasis [25]. As for the functional mechanism of circRNAs, increasing evidence suggested that circRNAs sponge miRNAs to carry out biological functions [7,8,13,16]. Liu et al. [26] reported that circIGHG directly sponge miR-142-5p to induce epithelial-to-mesenchymal transition, which promoted oral squamous cell carcinoma progression. Zhi et al. [27] identified that circ102049 significantly accelerated the invasion, migration, adhesion, and metastasis of colorectal cancer cells via sponging miR-761/miR-192-3p. Fang et al. [28] reported that downregulation of circRNA_0044516 inhibits gastric cancer cell proliferation via sponging mir-149. In our study, we identified that circRNA_0067934 could target miR-7 to regulate glioma cell proliferation and development. Similar to previous study [23], miR-7 was significantly down-regulated in human glioma tissues, moreover, the expression level of miR-7 was negatively associated with the expression level of circRNA_0067934. The results of dual-luciferase reporter assay suggested that circRNA_0067934 significantly targeted miR-7. Besides, the expression of miR-7 was significantly reduced by inhibiting circRNA_0067934 expression. Moreover, in the si-RNA and miR-7 inhibitors co-transfected system, the effect of down-regulated circRNA_0067934 was reversed due to function of miR-7 inhibitors, indicating that circRNA_0067934 can sponge miR-7 to influence the glioma cells’ proliferation, invasion, and migration.

As a highly conserved pathway, Wnt/β-catenin signaling pathway plays a crucial role in cells’ molecular functions, including differentiation, proliferation, migration, apoptosis, stem cell renewal, and genetic stability [29]. Wnt gene was initially identified by Nusse and Varmus in 1982 when they were studying oncogene by using mouse mammary tumor virus, and they initially named the gene INT1 [30]. Subsequently, INT1 gene was found to be the homologue of the Drosophila Wingless gene [31]. Then, a study reported that human INT1 gene was similar to mouse INT1, which indicated a highly conserved nature in various species [32]. After the discovery of several other activated genes [33], a hybrid name ‘WNT’ (Wingless-related integration site) was created to describe the INT1/Wingless family. The biological mechanism of WNT pathway was subsequently discovered, including a canonical pathway (involvement of β-catenin) and a non-canonical pathway [29]. β-catenin is a core cadherin protein complex, which can be activated by Wnt protein to activate Wnt/β-catenin signaling pathway [29]. Wnt/β-catenin pathway is involved in the development and progression of various cancers, such as gastric cancer [28], colorectal cancer [34], non-small cell lung cancer [35], melanoma [36], and glioma [21]. In the current study, we identified that circRNA_0067934 and miR-7 regulated the biological function of glioma cells by targeting the wnt/β-catenin pathway. Compared to the negative control group, the results of WB and IF revealed that wnt and β-catenin protein were down-regulated in the knockdown circRNA_0067934 group. Additionally, in the co-transfected system in which circRNA_0067934 and miR-7 were inhibited, the wnt and β-catenin protein levels were relatively up-regulated, indicating that the effect from down-regulating circRNA_0067934 was reversed. These results suggest that not only circRNA_0067934, but also miR-7 can regulate the wnt/β-catenin pathway.

## Conclusion

Our study indicated that the expression level of circRNA_0067934 was up-regulated in glioma tissues. Moreover, circRNA_0067934 can promote malignant behaviors of cells, including cell proliferation, invasion, and migration. Also, miR-7 functions as a molecular sponge of circRNA_0067934 and can regulate tumor progression by activating the Wnt/β-catenin pathway. The results of this study suggested that circRNA_0067934 could be a potential target for the treatment of glioma.

## Supplementary Material

Supplemental Material
