# Supplementary material for: Circular RNA circRNA_0067934 promotes glioma development by modulating the microRNA miR-7/ Wnt/β-catenin axis
Source: Bioengineered. 2022 Feb 25;13(3):5792–802. doi: 10.1080/21655979.2022.2033382 (PMC8973834; doi:10.1080/21655979.2022.2033382)
Supplement: Supplemental Material [file KBIE_A_2033382_SM5439.docx]

| **Table 1.** Basic characteristics of glioma patients. | | | | | | |
| --- | --- | --- | --- | --- | --- | --- |
| Number | Gender | Age | Tumor size | WHO | Relative expression of circ_0067932 | Relative expression of miR-7-5p |
| 1 | female | 54 | 7*8*5 | Ⅳ | 2.277 | 0.959 |
| 2 | male | 51 | 5*5*5 | Ⅳ | 1.694 | 0.975 |
| 3 | female | 63 | 3.5*3.5*2.5 | Ⅲ | 1.537 | 0.644 |
| 4 | female | 52 | 5*5*5 | Ⅱ | 1.134 | 0.573 |
| 5 | male | 48 | 5*5*5 | Ⅳ | 2.680 | 0.607 |
| 6 | female | 44 | 6*5*5 | Ⅳ | 1.714 | 0.711 |
| 7 | male | 43 | 6*6*8 | Ⅱ | 1.351 | 0.634 |
| 8 | male | 76 | 4*5*6 | Ⅳ | 1.478 | 0.375 |
|  |  |  |  |  |  |  |
